# Fourier-Domain Optical Coherence Tomographic Assessment of Changes in the Schlemm’s Canal of Nonglaucomatous Subjects After Keratoplasty

**DOI:** 10.3389/fphys.2021.716117

**Published:** 2021-12-02

**Authors:** Yujin Zhao, Yue Li, Jiaxu Hong, Qihua Le, Jianjiang Xu

**Affiliations:** ^1^Department of Ophthalmology and Visual Science, Eye and ENT Hospital, Shanghai Medical College, Fudan University, Shanghai, China; ^2^NHC Key Laboratory of Myopia (Fudan University), Laboratory of Myopia, Chinese Academy of Medical Sciences, Shanghai, China; ^3^Key Laboratory of Myopia, National Health and Family Planning Commission, Shanghai, China

**Keywords:** cornea, keratoplasty, Schlemm’s canal (SC), Fourier domain optical coherence tomography (FD-OCT), *in vivo* imaging

## Abstract

**Purpose:** This study aimed to evaluate the impact of keratoplasty on the *in vivo* anatomical structures in the Schlemm’s canal (SC) of nonglaucomatous subjects using Fourier-domain optical coherence tomography (FD-OCT).

**Methods:** Sixty-six nonglaucomatous eyes that underwent penetrating keratoplasty (PK), deep anterior lamellar keratoplasty (DALK), or triple surgery were enrolled in this prospective, comparative, observational study. The SC imaging was performed using FD-OCT before and after surgery in both the nasal and temporal quadrants. Patient demographics, SC parameters [e.g., cross-sectional area (CSA), meridional diameter of SC (MSC), sagittal diameter of SC (SSC), and circumference (CCF)], and the correlations between the variation of SC parameters and intraocular pressure (IOP) were analyzed.

**Results:** The mean age of all subjects was 40.27 ± 18.97 years. Among all cases, the nasal, temporal, and mean MSC significantly decreased on the first day after surgery and then increased at 1 week (*p* = 0.04, 0.017, and 0.01, respectively). Temporal CSA (tCSA), temporal MSC (tMSC), and temporal circumference (tCCF) after PK (*p* = 0.017, 0.020, and 0.018, respectively) and nasal MSC (nMSC) after DALK (*p* = 0.025) decreased significantly on the first day after surgery. The shift in mean IOP was significantly correlated with the changes in tMSC (*r* = 0.341, *p* = 0.003) and CCF (*r* = 0.207, *p* = 0.048).

**Conclusion:** SC had significant *in vivo* morphological changes in the early period after keratoplasty in nonglaucomatous eyes, accompanied with elevation of IOP. Early intervention might be necessary to prevent secondary glaucoma early after keratoplasty.

## Introduction

Since the first description of the technique in 1994, anterior segment optical coherence tomography (AS-OCT) has become increasingly important in clinical practice (Izatt et al., 1994; Ang et al., 2018). Due to a low risk of contact infection, a high imaging resolution, and adequate scanning depth, this procedure is one of the best choices for observations of early postoperative patients when compared with ultrasound biomicroscopy (UBM) (Kanellopoulos and Asimellis, 2013; Zhao et al., 2016; Qi et al., 2020). Specifically, Fourier-domain OCT (FD-OCT) has the advantage of high scanning speed and image quality for a wide range of clinical applications (Mayer et al., 2010; Told et al., 2016; Kreuz et al., 2018; Napoli et al., 2020). The anterior segment CASIA SS-1000 OCT (Tomey Corp., Nagoya, Japan) is a Fourier-domain, swept-source designed OCT that is reported to be suitable for studying the anatomical structures of aqueous outflow system, including Schlemm’s canal (SC). It uses a light source with a wavelength of 1,310 nm and has a high scanning speed of 30,000 A-scans per second and 256 B-scans over the scanning area. The axial resolution is 10 μm or less, and transverse resolution is 30 μm. The scanning area is as high as 16 × 16 mm (horizontal and vertical) with a depth of 6 mm (Kagemann et al., 2010; Hong et al., 2013; Angmo et al., 2016; Qi et al., 2020).

As a significant component of anterior chamber angle structures and a critical part in the conventional aqueous humor outflow pathway, the SC was reported to generate approximately 49–90% of the total outflow resistance in healthy eyes (Chen et al., 2019; Wang et al., 2020). Specifically, the inner wall of the SC is the critical structure that directly relates to elevated IOP resulting from increasing stiffness and flow resistance in glaucomatous eyes (Vahabikashi et al., 2019). Notably, the morphology of SC tends to vary with changes in intraocular pressure (IOP) in different pathophysiological conditions (Zhao et al., 2016; Li et al., 2017). However, the keratoplasty-related changes in the morphology of SC are still unknown.

With an incidence rate of 5.5–31%, ocular hypertension (OHT) or secondary glaucoma (SG) in the early postoperative period after penetrating keratoplasty (PK) may lead to endothelial dysfunction and graft failure (Karadag et al., 2010; Ali et al., 2011; Kornmann and Gedde, 2016). Changes of anterior chamber parameters, including anterior chamber depth and volume and iridocorneal angle, were detected after keratoplasty (Gatzioufas et al., 2012; Ort et al., 2017), yet the SC was not explored. Therefore, we hypothesized that keratoplasty-induced anterior chamber structural changes, especially in the morphology of SC, could cause early postoperative OHT.

As a result, we aimed to investigate the morphological alterations of the SC in the immediate and early periods after keratoplasties and to explore their correlations with changes in IOP in nonglaucomatous eyes.

## Materials and Methods

This prospective, comparative, observational study was approved by the Eye & ENT Hospital of Fudan University. All subjects provided informed consents and all the procedures adhered to the tenets of the Declaration of Helsinki.

### Participants

This study was conducted from August 2018 to December 2020 at the Eye & ENT Hospital of Fudan University, Shanghai, China. Comprehensive ophthalmic examinations, including Snellen best-corrected visual acuity (BCVA), slit-lamp biomicroscopy, IOP (Goldmann applanation tonometry), and AS-OCT scan, were conducted before and on each follow-up at 1 day and 1 week after surgery. IOP and AS-OCT examinations were performed in the morning (8:00–12:00). Patients with history of concurrent glaucoma or ocular trauma were excluded. High axial myopia was also excluded because it affected the morphology of SC (Chen et al., 2018). An accurate IOP measurement was challenging to obtain in keratoconus with acute corneal hydrops using Goldmann applanation tonometry (and these cases were excluded).

### Surgical Procedures

Retrobulbar and peribulbar anesthesia were used for all subjects. The PK and deep anterior lamellar keratoplasty (DALK) procedures were the same as in our previous study (Zhao et al., 2020). The triple surgery, which is composed of PK, extracapsular cataract extraction (ECCE), and intraocular lens (IOL) implantation, was chosen after careful consideration of multiple factors, including age, depth of lesion, and choice of patients. The pupil was dilated with 2.5% phenylephrine at the beginning of the operation. After excision of the original cornea of patients, continuous curvilinear capsulorhexis (CCC) was completed using forceps. The lens cortex was aspirated by an irrigation-aspiration tip, which was connected to the phacoemulsification console. IOL was then inserted into the capsular bag as the donor graft was sutured with 10 interrupted sutures. Interrupted 10-0 nylon nonabsorbable surgical sutures (USIOL, Inc., Lexington, KY, United States) were used to secure the graft to the host. Topical TobraDex eyedrops (Alcon, Rijksweg, Belgium) four times a day were used for at least 3 months after surgery.

### Measurement of Intraocular Pressure

Goldmann applanation tonometry (Haag-Streit, Bern, Switzerland) was used for preoperative and postoperative (at 1 day and 1 week) IOP assessment. Measurement was performed after instillation of 0.04% Benoxil eyedrops (Santen Pharmaceutical Co., Ltd.) and positioning the scale of the tonometer on 10 mm Hg. Only one measurement was taken with no separate reading, and the operator was masked to the AS-OCT data. Each IOP measurement was conducted by only one experienced operator (T.L.J.) to reduce the operational difference. The existing equipment in our hospital was not modified by any advanced prisms for adjustment.

### Anterior Segment Optical Coherence Tomography Imaging

Anterior segment optical coherence tomography (CASIA SS-1000, Tomey Corp.) scans were all performed under dark room condition. With ANGLE (HD) mode, the subjects were asked to focus on peripheral fixation lights and scans were centered on the corneoscleral sites of both the nasal and temporal quadrants (at 3 and 9 o’clock positions) according to previous reports (McKee et al., 2013; Gao et al., 2017). Three consecutive scans of each side were acquired. Each scan comprised 128 frames spaced 1.4 degrees apart. All scans were performed by one experienced ophthalmologist who was masked to the clinical status of the subjects and scanning parameters and were same for all patients.

### Measurement of Schlemm’s Canal Morphology Parameters

**FIGURE 1:**
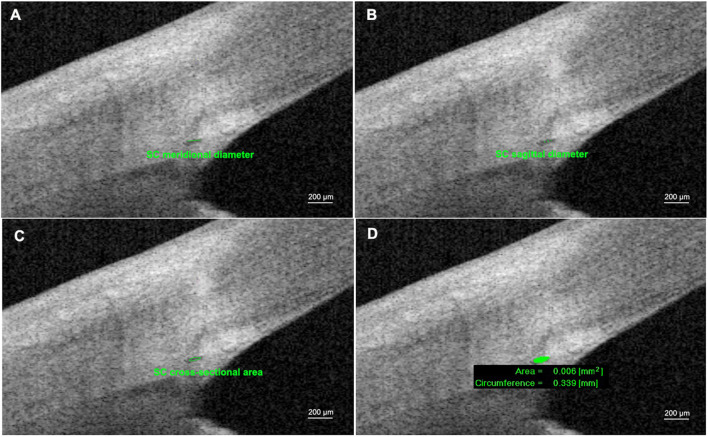
Measurements of Schlemm’s canal (SC) using anterior segment optical coherence tomography. **(A)** SC meridional diameter was the distance of the longest axis of SC measuring from the posterior to the anterior SC end point. **(B)** SC sagittal diameter was defined as the distance between the superior and inferior outline of SC lumen (average of three measurements). **(C,D)** SC cross-sectional area (CSA) and circumference of SC (CCF) were obtained by automatic calculation process after manually pointing out the boundary of SC lumen.

Images with adequate quality were chosen for the following analysis. The SC parameters included sagittal diameter of SC (SSC), meridional diameter of SC (MSC), cross-sectional area of SC (CSA), and circumference of SC (CCF). SC sagittal diameter was defined as the distance between the superior and inferior outline of the SC lumen (average of three measurements). MSC was the distance of the longest axis of SC measuring from the posterior to the anterior SC end point (Qi et al., 2020; Figure 1). All the images were taken after 200× magnification for best visualization. Three frames with clear visualization of SC from each image were selected for the following measurements. The CSA and CCF were obtained by automatic calculation processes after manual delineation of SC by two separate operators. Consensus was reached when disagreements regarding the delineation occurred. All parameters of SC morphology were acquired as the average values by nine repeated measurements from three consecutive images.

The SC parameters in both the nasal and temporal quadrants were recorded as nCSA, nSSC, nMSC, nCCF, tCSA, tSSC, tMSC, and tCCF. To evaluate the overall situations, combined SC parameters were acquired by averaging nasal and temporal counterparts from one examined eye and recorded as CSA, SSC, MSC, and CCF. Excellent repeatability and reproducibility in measurements of SC using this device were demonstrated in previous studies (Chen et al., 2018; Qi et al., 2020).

### Statistical Analysis

All BCVA values, including the Count Finger (CF) and Hand Move (HM), were transferred into the logarithm of the minimum angle of resolution BCVA (LogMAR BCVA) before analysis (Schulze et al., 2006; Lange et al., 2009). Qualitative variables were presented as arithmetic mean [standard deviation (SD)]. Statistical analysis was performed using SPSS version 22.0 (IBM Corp., Armonk, NY, United States). Analysis of variance (ANOVA) test and Kruskal–Wallis test were performed to compare preoperative and postoperative parameters. Wilcoxon signed-rank test and paired *t*-test were used to compare the parameters before and at the 1 day time point after three different surgeries. The correlations between the change of IOP and SC parameters were analyzed using Spearman’s correlation. To evaluate the reliability and repeatability between multiple measurements of the same eye, the intraclass correlation coefficient (ICC) was used. The ICC measures the proportion of total variability in measurements contributed by variability in manual measurements among three consecutive scans and was determined using the random-effects mixed model. A *p*-value < 0.05 was considered statistically significant.

## Results

**FIGURE 2:**
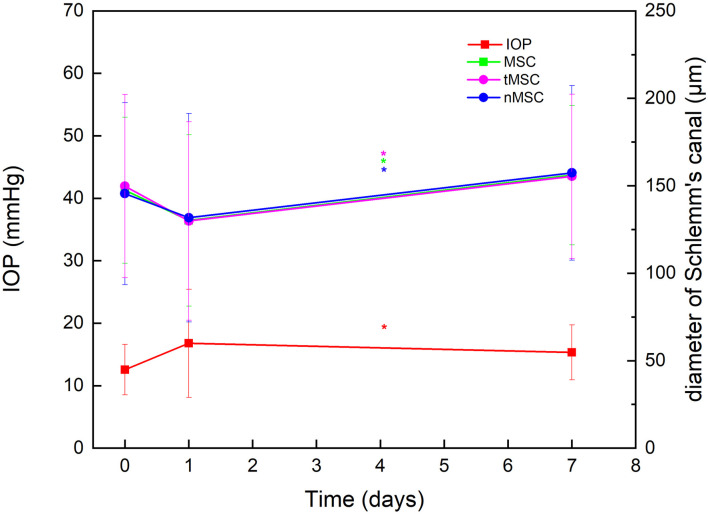
Plotting line showing the significantly opposite variation trends of intraocular pressure (IOP) against nasal meridional diameter of Schlemm’s canal (nMSC), temporal meridional diameter of Schlemm’s canal (tMSC) and MSC before surgery, on day 1 after surgery, and at 1 week after surgery. *Indicates statistical difference between date groups.

**FIGURE 3:**
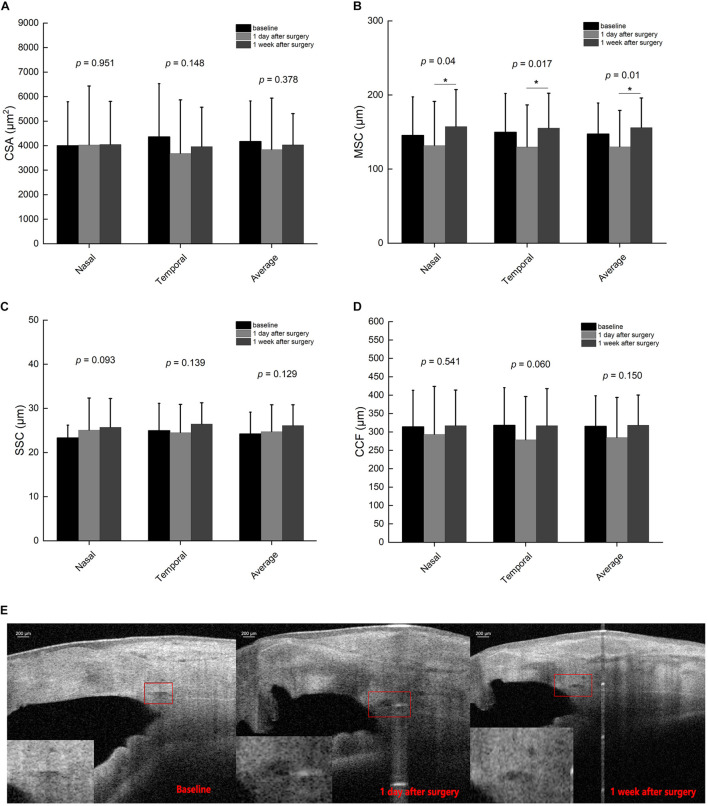
Schlemm’s canal (SC) parameters including cross-sectional area (CSA) **(A)**, sagittal diameter of SC (SSC) **(C),** and circumference (CCF) **(D)** did not show significant differences among preoperative and postoperative time points, except meridional diameter of SC (MSC) **(B)**. The SC morphology varied at different time points before and after surgery **(E)**. *Indicates statistical difference between date groups.

A total of 66 eyes from 66 patients (44 males and 22 females) were enrolled. The mean age was 40.27 (18.97) years (range, 14–79). Details of indication types are presented in Table 1. Due to the small sample size, data in the triple surgery group were excluded from merged statistics (Table 2). Among postoperative SC parameters, nMSC, tMSC, and MSC significantly decreased on the first day, then increased at 1 week (*p* = 0.032, 0.021, and 0.024, respectively). IOP showed concomitant variations in which it increased from 12.33 (4.06) to 16.84 (8.61) mm Hg at the first day (*p* = 0.003), then dropped to 15.25 (4.39) mm Hg at 1 week (before vs. 1 week after surgery, *p* = 0.000) (Figure 2). Details of comparisons between preoperative and postoperative parameters are listed in Table 2 and Figure 3.

**TABLE 1:** Distribution of preoperative indications and surgery types.

**Surgery types**	**Numbers**	**Indications**
PK	31	8 corneal stromal dystrophies, 7 KCN, 7 corneal leucoma, 5 corneal graft failure, 4 endothelial decompensation
DALK	30	24 KCN, 4 corneal leucoma, 2 corneal stromal dystrophies
Triple surgery	5	2 corneal leucoma, 2 corneal stromal dystrophies, and 1 endothelial decompensation

*PK, penetrating keratoplasty; DALK, deep anterior lamellar keratoplasty; KCN, keratoconus.*

**TABLE 2:** Comparisons between preoperative and postoperative parameters.

	**Baseline**	**1 day**	**1 week**	***p***
logMAR BCVA	1.54 (0.55)	0.95 (0.49)	1.04 (0.49)	**0.000**
IOP (mmHg)	12.55 (4.03)	16.79 (8.64)	15.33 (4.37)	**0.000**
nCSA (μm^2^)	4000.00 (1794.09)	4030.77 (2404.22)	4052.63 (1756.83)	0.951
nMSC (μm)	145.52 (52.01)	131.72 (59.61)	157.39 (49.90)	**0.04**
nSSC (μm)	23.33 (2.88)	25.09 (7.25)	25.73 (6.51)	0.093
nCCF (μm)	314.22 (98.96)	293.51 (130.19)	317.21 (96.76)	0.541
tCSA (μm^2^)	4363.63 (2167.14)	3681.82 (2184.98)	3965.52 (1600.06)	0.148
tMSC (μm)	149.74 (52.37)	129.89 (56.73)	155.41 (46.94)	**0.017**
tSSC (μm)	24.96 (6.21)	24.50 (6.39)	26.46 (4.80)	0.139
tCCF (μm)	318.32 (102.19)	278.71 (117.54)	317.24 (100.37)	0.06
CSA (μm^2^)	4174.24 (1646.66)	3840.91 (2096.08)	4035.09 (1274.26)	0.378
MSC (μm)	147.42 (41.78)	130.17 (48.96)	156.11 (39.82)	**0.01**
SSC (μm)	24.22 (4.92)	24.74 (6.07)	26.12 (4.71)	0.129
CCF (μm)	315.61 (82.77)	284.95 (108.82)	318.52 (81.83)	0.150

*LogMAR BCVA, logarithm of the minimum angle of resolution best-corrected visual acuity; IOP, intraocular pressure; n, nasal; t, temporal; CSA, cross-sectional area; MSC, meridional diameter of Schlemm’s canal; SSC, sagittal diameter of Schlemm’s canal; CCF, circumference. Analysis of variance (ANOVA) test and Kruskal–Wallis test were performed for these parameters. All quantitative values were given as mean (standard deviation), *p* < 0.05, and bold values indicate statistical significance.*

**FIGURE 4:**
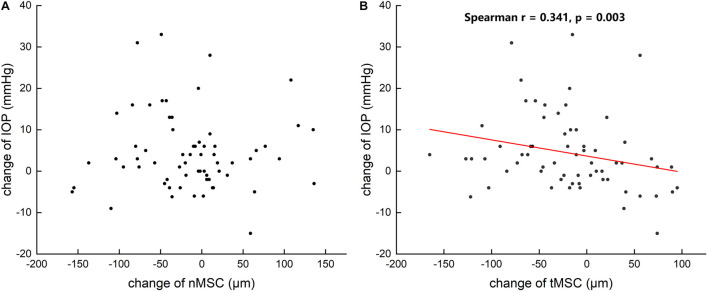
Scattergrams showing the correlations between the changes in preoperative and postoperative intraocular pressure (IOP) and the changes in the nasal meridional diameter of Schlemm’s canal (nMSC) **(A)** and the temporal meridional diameter of Schlemm’s canal (tMSC) **(B)** in all subjects.

**FIGURE 5:**
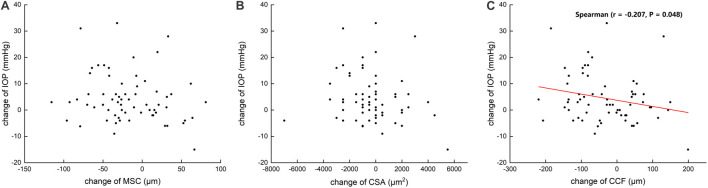
Scattergrams showing the correlations between the changes in preoperative and postoperative intraocular pressure (IOP) and the changes in the meridional diameter of Schlemm’s canal (MSC) **(A)** and cross-sectional area (CSA) **(B)** and circumference (CCF) **(C)** in all subjects.

Table 3 summarizes that at 1 day after operation, tCSA, tMSC, and tCCF decreased significantly (*p* = 0.017, 0.020, and 0.018, respectively) in the PK group and nMSC decreased (*p* = 0.025) in the DALK group. No statistical differences were observed in the triple surgery group. Correlation analysis showed the augmentation of IOP at the first day after surgery was negatively correlated with the changes in tMSC (*r* = -0.341, *p* = 0.003) and CCF (*r* = -0.207, *p* = 0.048) for all subjects (Figures 4, 5).

**TABLE 3:** Comparisons between preoperative and postoperative (1 day) parameters of different surgeries.

	**PK**		***p***	**DALK**		***p***	**Triple surgery**		***p***
	**Baseline**	**1 day**		**Baseline**	**1 day**		**Baseline**	**1 day**	
LogMAR BCVA	1.73 (0.47)	0.91 (0.54)	**0.000**	*1.28 (0.52)*	0.96 (0.41)	**0.001**	1.89 (0.65)	1.18 (0.64)	0.109
IOP (mmHg)	14.20 (3.95)	17.74 (10.05)	0.102	10.4 (3.22)	15.90 (6.86)	**0.001**	15.2 (2.59)	16.2 (10.11)	0.686
nCSA (μm^2^)	3903.23 (2071.31)	3838.71 (2608.92)	0.625	4103.45 (1496.30)	4310.34 (2331.51)	0.614	4000.00 (1870.83)	3600.00 (1516.58)	0.581
nMSC (μm)	131.13 (48.18)	132.61 (62.91)	0.799	160.72 (53.82)	131.79 (59.01)	**0.025**	146.60 (48.07)	125.80 (52.38)	0.480
nSSC (μm)	24.02 (6.09)	23.76 (7.22)	0.850	22.60 (4.64)	26.23 (7.60)	**0.009**	24.03 (3.49)	26.80 (4.27)	0.112
nCCF (μm)	292.77 (93.18)	282.84 (140.32)	0.388	340.21 (94.49)	310.14 (124.10)	0.151	296.40 (142.18)	263.20 (108.07)	0.618
tCSA (μm^2^)	4032.26 (2373.24)	3129.03 (1892.83)	**0.017**	4766.67 (1959.65)	4166.67 (2450.66)	0.135	4000.00 (2000.00)	4200.00 (1643.17)	0.778
tMSC (μm)	143.90 (48.41)	116.19 (50.21)	**0.020**	149.73 (51.19)	138.57 (60.33)	0.241	186.00 (78.04)	162.80 (60.22)	0.521
tSSC (μm)	23.40 (6.44)	22.88 (4.70)	0.991	26.58 (5.52)	25.70 (7.70)	0.584	24.87 (7.40)	27.33 (5.08)	0.389
tCCF (μm)	311.32 (107.01)	251.94 (111.45)	**0.018**	326.03 (103.50)	302.10 (123.18)	0.237	315.40 (72.85)	304.40 (104.30)	0.673

*PK, penetrating keratoplasty; DALK, deep anterior lamellar keratoplasty; LogMAR BCVA, logarithm of the minimum angle of resolution best corrected visual acuity; IOP, intraocular pressure; n, nasal; t, temporal; CSA, cross-sectional area; MSC, meridional diameter of Schlemm’s canal, SSC, sagittal diameter of Schlemm’s canal; CCF, circumference. Wilcoxon signed-rank test and paired *t*-test were performed for these parameters. All quantitative values were given as mean (standard deviation), *p* < 0.05, and bold values indicate statistical significance.*

The ICC values reflected excellent measurement repeatability. The ICC values of SC parameters were as follows: SSC = 0.90, MSC = 0.88, CSA = 0.95, and CCF = 0.90.

## Discussion

Based on previous reports (Allingham et al., 1996; Kagemann et al., 2014; Rubinstein et al., 2019), the level of IOP is usually inversely related to the size of the SC. A similar trend of IOP and morphology of SC was found in this study. The values of MSC on both the nasal and temporal quadrants initially decreased after keratoplasty and then recovered at 1 week. Meanwhile, IOP first increased and then decreased, which was opposite to the morphological change in SC.

Corneal surgeons particularly emphasize IOP elevation due to its negative effect on graft survival rate. However, it is not easy to monitor IOP in the early period after keratoplasty (Fini et al., 2017). Few studies have been published before this study investigating the relationship between the anterior chamber structures and IOP in the immediate and early period after keratoplasty. Researchers (Gatzioufas et al., 2012) found that increased IOP after excimer laser PK was significantly correlated with the depth (*p* < 0.001) and volume (*p* = 0.03) of the anterior chamber. However, the morphological change in SC after keratoplasty was not described.

The inherent properties of the SC can result in its deformability. SC has dual anatomical properties of being both lymphatic and blood vessels. The combination of incomplete basement membrane, tight connections among endothelial cells, and vertical force direction on the endothelial cells allows a greater possibility of deformation and collapse of the SC (Bhatt et al., 1995; Ramos et al., 2007; Watanabe et al., 2009).

Inner-ocular surgery may disrupt the blood-aqueous barrier and induce postoperative inflammation (Hori et al., 2019). This is generally characterized by inflammatory mediator release, blood vessels dilation, and contraction or relaxation of lymphatic vessels in different tissues (von der Weid, 2001; Krüger et al., 2019). We speculated that the quasi-lymphatic characteristic of the SC might contract with the postoperative inflammation status, which manifested as a reduction in the diameter and circumference of SC. Additionally, increased blood flow at the cornea and anterior uvea after surgery (Basu et al., 1978; Avetisov et al., 2016) might magnify the volume of reflux liquid and then condense the SC.

Although the trend of MSC change was obvious in the merged statistical analysis, comparisons among different keratoplasty types showed that PK led to more obvious morphological alterations compared with DALK on the first day after surgery. It was proved that the replacement of lamellar thickness of the corneal tissue of recipients causes less traction power to the corneoscleral tissue and anatomical changes to the anterior chamber angle when compared with full-thickness corneal tissue transplantation (Ort et al., 2017). DALK preserves the intact Descemet’s membrane (DM) and endothelium. The mechanical continuity and stiffness not only reduces the pressure gradient between the interior of the eye and the ambient atmosphere but also helps to partially maintain the biomechanical properties of itself and the overlying stroma (Schulze et al., 2006; Ali et al., 2016; Jiang et al., 2017). This might be the primary reason for fewer SC parameter alterations after DALK compared with after PK. Interestingly, morphological changes in SC were observed only on the temporal side in PK group and only on the nasal side in DALK group. Larger size of SC on the nasal side (Kagemann et al., 2010), different residual stromal thickness in PK and DALK procedures, and negative correlations between corneal deformation and residual stromal thickness (Li et al., 2021) may cause this phenomenon in some unknown and complicated ways that need further exploration.

Our study has several limitations. First, the sample size of the triple surgery group was limited and this constricted deeper understanding of the effect on SC from multiple operations. Second, although the Goldmann applanation tonometer is considered the “gold standard” of IOP measurement, it might be influenced by corneal biomechanics (Rubinfeld et al., 1998; Asaoka et al., 2015). Swollen corneal tissue in the early and immediate period after keratoplasty may overrate the actual IOP (Cairns et al., 2019). The bias in measurements is difficult to avoid since our equipment was not modified by any advanced prisms for adjustment.

Our study found that in nonglaucomatous eyes, MSC significantly decreased after corneal transplantations on day 1 (postoperatively) and then recovered after 1 week. Accordingly, IOP increased initially and then decreased to normal. PK procedures may lead to more morphological changes in the SC compared with DALK. Further studies with a larger sample size are required to demonstrate the variations in SC morphology during the immediate and early period after surgery in order to provide more evidence for postoperative medication and prevent OHT or SG.

## Data Availability Statement

The original contributions presented in the study are included in the article/supplementary material, further inquiries can be directed to the corresponding author.

## Ethics Statement

The studies involving human participants were reviewed and approved by the Eye & ENT Hospital of Fudan University. The patients/participants provided their written informed consent to participate in this study.

## Author Contributions

JH, JX, and YZ designed the experiment. YZ and YL conducted the experiment. YZ wrote the initial draft after analyzing the data. QL and JX revised the manuscript and made the final version. All authors discussed the analyzed data and the interpretations.

## Conflict of Interest

The authors declare that the research was conducted in the absence of any commercial or financial relationships that could be construed as a potential conflict of interest.

## Publisher’s Note

All claims expressed in this article are solely those of the authors and do not necessarily represent those of their affiliated organizations, or those of the publisher, the editors and the reviewers. Any product that may be evaluated in this article, or claim that may be made by its manufacturer, is not guaranteed or endorsed by the publisher.
